# Integrating biokinetics and in vitro studies to evaluate developmental neurotoxicity induced by chlorpyrifos in human iPSC-derived neural stem cells undergoing differentiation towards neuronal and glial cells

**DOI:** 10.1016/j.reprotox.2020.09.010

**Published:** 2020-12

**Authors:** Emma Di Consiglio, Francesca Pistollato, Emilio Mendoza-De Gyves, Anna Bal-Price, Emanuela Testai

**Affiliations:** a Istituto Superiore di Sanità, Environment and Health Department, Mechanisms, Biomarkers and Models Unit, Rome, Italy; b European Commission, Joint Research Centre (JRC), Ispra, Italy

**Keywords:** Chlorpyrifos, Biokinetics, Developmental neurotoxicity, Repeated exposure, Human iPSC-derived neural stem cells

## Abstract

For some complex toxicological endpoints, chemical safety assessment has conventionally relied on animal testing. Apart from the ethical issues, also scientific considerations have been raised concerning the traditional approach, highlighting the importance for considering real life exposure scenario. Implementation of flexible testing strategies, integrating multiple sources of information, including in vitro reliable test methods and in vitro biokinetics, would enhance the relevance of the obtained results. Such an approach could be pivotal in the evaluation of developmental neurotoxicity (DNT), especially when applied to human cell-based models, mimicking key neurodevelopmental processes, relevant to human brain development.

Here, we integrated the kinetic behaviour with the toxicodynamic alterations of chlorpyrifos (CPF), such as in vitro endpoints specific for DNT evaluation, after repeated exposure during differentiation of human neural stem cells into a mixed culture of neurons and astrocytes. The upregulation of some cytochrome P450 and glutathione S-transferase genes during neuronal differentiation and the formation of the two major CPF metabolites (due to bioactivation and detoxification) supported the metabolic competence of the used in vitro model. The alterations in the number of synapses, neurite outgrowth, brain derived neurotrophic factor, the proportion of neurons and astrocytes, as well as spontaneous electrical activity correlated well with the CPF ability to enter the cells and be bioactivated to CPF-oxon. Overall, our results confirm that combining in vitro biokinetics and assays to evaluate effects on neurodevelopmental endpoints in human cells should be regarded as a key strategy for a quantitative characterization of DNT effects.

## Highlights

• Human iPSC-derived NSCs undergoing differentiation possess some metabolic competence.
• CPF entered the cells and was biotrasformed into its two main metabolites (CPFO and TCP).
• After repeated exposure, very limited bioaccumulation of CPF was observed.
• Treatment with CPF decreased neurite outgrowth, synapse number and electrical activity.
• Treatment with CPF increased BDNF levels and the percentage of astrocytes.

## Graphical abstract

**Figure:**
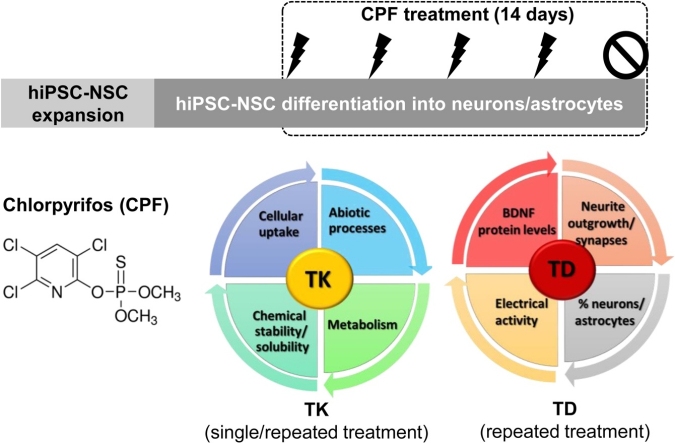

## 1 Introduction

In recent years, there has been an increased awareness of the usefulness of data coming from approaches other than animal testing for chemical safety assessment [[1], [2], [3]]. Flexible testing strategies based on non-animal systems, resembling- as much as possible- human biology, are needed for specific endpoint evaluation, both in the regulatory context and in biomedical research, including in vitro methods of high relevance to human biology conducted with a robust study design [4].

On top of that, the need to define a reliable reference point from in vitro concentration-response data, thus identifying safe exposure levels for humans is a key aspect in the regulatory context [5,6]. With the application and improvement of novel or existing *in silico* approaches aimed to support in vitro to in vivo extrapolation (QIVIVE) and Physiologically based pharmacokinetic (PBPK) models [[7], [8], [9], [10], [11]], some proofs of concept have been developed [10,12,13]. On this basis, in vitro concentrations able to give a specific response can be regarded as “internal biologically effective doses” in vivo.

Most of the in vitro toxicity endpoints are currently related to the nominal concentration of the chemical added at the beginning of testing. However, this is often a very rough estimation of real cell exposure and biologically effective in vitro concentrations [[14], [15], [16]]. Indeed, possible variations of test item concentration over time can be related to chemical reactions or limited stability, adsorption to plastic, binding to proteins and lipids present in cell culture medium or intracellular accumulation, especially in repeated-dosing studies [[17], [18], [19]]. These alterations may change the actual bioavailability of a compound, affecting its final adverse outcome [20]: therefore the assessment of in vitro kinetics is a pivotal element, especially for compounds that undergo bioactivation [18,19].

**Fig. 1:**
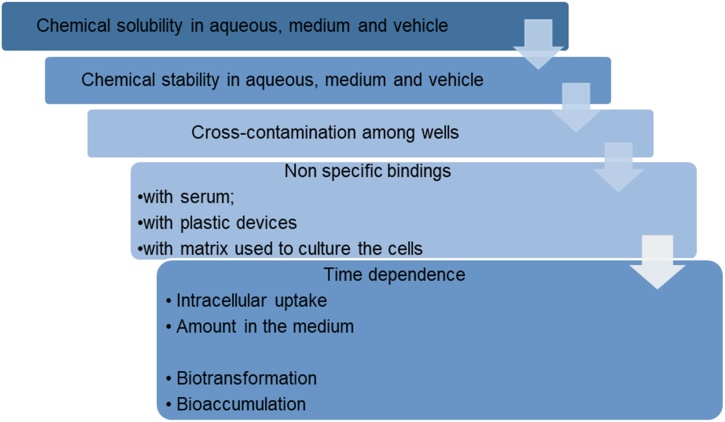
Step-wise procedure for the determination of chemical cellular bioavailability.

A step-wise procedure to characterise in vitro biokinetics was established within the European project PREDICT-IV (FP7-HEALTH program) [19,[21], [22], [23], [24]], and has been recently endorsed in the OECD Guidance Document on Good In Vitro Method Practices (GIVIMP) [25]. A schematic description of this procedure is presented in Fig. 1. The characterization of the in vitro system following GIVIMP principles and evaluation of in vitro biokinetics is instrumental to improve credibility of in vitro toxicity testing and development of related dose-response metrics, leading to better mechanistic understanding of the pathways mediating adverse effects [26,27].

In recent years, developmental neurotoxicity (DNT) due to exposure to environmental contaminants has caused serious concern. Indeed, the developing brain is particularly vulnerable [[28], [29], [30], [31]], compared to the adult nervous system for several reasons. For instance, the blood brain barrier (BBB) is not completely developed at least until 6 months after birth [30], facilitating chemical entrance into the foetal/neonatal brain [32]; a wide range of dynamic complex cellular and molecular processes occurring during brain development [[33], [34], [35], [36], [37]], makes the foetal/neonatal brain susceptible to chemical exposure.

Despite this, DNT testing is not a mandatory requirement for the safety assessment of regulated products both in Europe and in the USA, being only performed as higher tiered tests, when triggered by structure activity alerts or evidence of neurotoxicity observed in standard in vivo developmental or reproductive toxicity studies (e.g., OECD Test Guidelines 426 and 443) [[38], [39], [40], [41]].

To date, some DNT studies, mainly on pesticides, have been published [42,43], and around 120 chemicals have been recognized as clear developmental neurotoxicants [[44], [45], [46]], using in vivo rodent studies that are costly, lengthy, requiring a high number of animals [[47], [48], [49]], and therefore rarely performed. The possible interspecies differences and data extrapolation to humans have hampered risk assessment and regulatory decision-making [38].

On the other hand, a wide range of well-characterized in vitro test systems is available, but none of them has been currently accepted for regulatory purposes. The use of human-derived in vitro models is preferable over animal-derived cells, to avoid interspecies differences, reduce uncertainties in results extrapolation, and improve prediction of human toxicity [39,50,51]. Human induced pluripotent stem cell (hiPSC)-derived neural stem cells (NSCs), differentiated into neuronal and glial cells are considered suitable, since they recapitulate key neurodevelopmental processes [52]. Anchoring DNT-relevant in vitro assays to key events (KEs) described in DNT-relevant adverse outcome pathways (AOPs) (i.e., AOP12 [53]; AOP13 [54]; AOP42 [55]; AOP54 [56]; AOP134 [57]; and [[58], [59], [60]]) leading to the same adverse outcome (AO) (i.e., cognitive deficit/learning and memory impairments in children) has been recently proposed as an effective strategy to evaluate DNT potential of different chemicals [52,59].

Recently, the creation of a battery of in vitro DNT test methods based on the use of human neuronal and glial cells, together with available in vivo and epidemiological data and in silico approaches has been envisaged as a meaningful approach to improve current DNT testing strategy, support the development of Integrated Approaches to Testing and Assessment (IATA) designed in a fit-for-purpose manner, serving different regulatory purposes (e.g., chemical prioritization for further screening, hazard identification and characterization or risk assessment), reducing time and costs [61,62].

However, DNT in vitro testing strategies cannot be accurately applied without considering the key element of the evaluation of metabolic competence of selected in vitro test systems, in order to understand whether (and how) chemicals are biotransformed by the cell model. Human embryonic stem cell-derived neocortical organoids have been shown to express certain cytochrome P450 (CYP) isoforms (e.g., CYP-3A5) [63], showing some metabolic competence. However, up to now, no studies have specifically reported on the expression of CYP and glutathione S-transferase (GST) enzymes in hiPSC-derived mixed neuronal and glial cultures.

In the present study, to our knowledge for the first time, the biokinetics and the effects of Chlorpyrifos (CPF), an organophosphate pesticide, were tested in hiPSC-NSCs undergoing differentiation toward neurons and astrocytes. In addition we characterised our cell model for the expression of those CYPs known to catalyse CPF desulfuration into the phosphate triester chlorpyrifos-oxon (CPFO), a potent serum and brain acetylcholinesterase (AChE) inhibitor, as well as CPF dearylation resulting in 3,5,6-trichloro-2-pyridinol (TCP) and diethyl thiophosphate (DETP), the two main detoxification products [64]. We measured both CYP and GST expression before and after 14-day treatment with CPF, we assessed the in vitro kinetics of CPF and its metabolites, correlating toxicodynamic outcomes with the actual cellular exposure in a concentration repeated regimen, and analysed the concentration-dependent effects of CPF on neurite outgrowth, the number of synapses, BDNF protein levels, the relative proportion of neurons and astrocytes, and the generation of spontaneous electrical activity, which is considered a reliable functional readout for neuronal cell evaluation.

## 2 Materials and methods

### 2.1 Chemicals and reagents

CPF (purity 98.8 %) was obtained from Chem Service (West Chester, PA), CPF-oxon (CPFO) and TCP (purity 99.5 %) were purchased from Sigma-Aldrich (Merck Life Science S.r.l. Milan, Italy). IMR90 hiPSC-neural stem cells (NSCs) were maintained in expansion in standard Matrigel® coated flasks (Corning) in the presence of DMEM/F12 medium supplemented with B27 (without vitamin A) and N2 supplements (all from ThermoFisher), Heparin Grade I-A (Sigma-Aldrich, 2 μg/mL), Non-Essential Amino Acids, penicillin/streptomycin (50 U/mL final concentration) and l-Glutamine (2 mM final concentration), and completed with basic fibroblast growth factor (bFGF, 10 ng/mL), epidermal growth factor (EGF, 10 ng/mL) and brain derived neurotrophic factor (BDNF, 2.5 ng/mL) (all from ThermoFisher). NSCs were differentiated into a mixed culture of neurons and astrocytes on poly-d-lysine (PDL)-96-well plates (Corning) coated with Matrigel® Growth Factor Reduced Matrix (Corning), in the presence of Neurobasal medium supplemented with B27 (with vitamin A) and N2, penicillin/streptomycin (50 U/mL final concentration) and l-Glutamine (2 mM), and completed with BDNF (2.5 ng/mL), glial cell-derived neurotrophic factor (GDNF, 1 ng/mL) and laminin (1 μg/mL) (all from ThermoFisher). For CPF and metabolite quantification, analytical grade chemicals were obtained from commercially available sources. The Milli-Q water purification system (Millipore, Merck KGaA, Darmstadt, Germany) was used to obtain deionised water.

### 2.2 Human induced pluripotent stem cell (hiPSC)-derived neural stem cells (NSCs) differentiated into a mixed culture of neurons and astrocytes

**Fig. 2:**
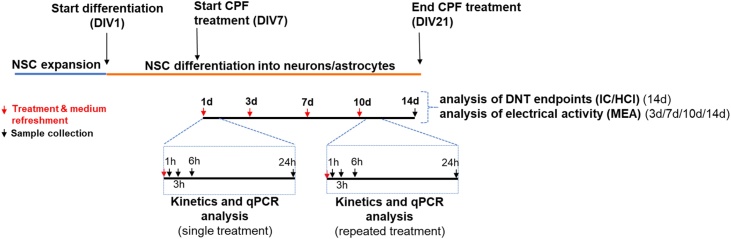
Scheme summarizing the experimental design, CPF treatments and performed analyses.

Neural stem cells (NSCs) were originally derived from IMR90-hiPSCs (generated and kindly provided by Prof Marc Peschanski, I-Stem, France), and differentiated to obtain a mixed culture of neurons and astrocytes, as detailed in [65]. Briefly, NSCs were passaged with Trypsin, plated onto 96 well PDL-coated plates coated with reduced growth factor matrigel-coated at a density of 7000 cells/well (150 μL/well) (i.e., 21,000 cells/cm^2^), and differentiated for 21 days in vitro (DIV) (Fig. 2), as already described [65,66], refreshing both medium and treatments twice a week. After 21 DIV, culture was characterized by ∼60 % neurons (mainly glutamatergic), 18–24 % astrocytes, along with some nestin^+^ NSCs (∼20 %), as previously described [65,66].

After 7 DIV, cells were treated for 14 days in the presence of CPF. In our previous study [52], among three in vitro tested concentrations, i.e., 37.1 μM (equal to IC_20_), 21 μM (equal to IC_5_), and 0.37 μM (equal to IC_20_/100), IC_5_ resulted as the lowest concentration inducing a statistically significant variation of synaptogenesis [52]. To carry out the analysis of gene expression (for CYP and GST genes, see Section 2.3) and electrical activity (by MEA, see Section 2.5.2), we considered 21 μM (IC_5_) CPF. To set-up concentration response curves for the analysis of some of the selected DNT endpoints (synaptogenesis, neurite outgrowth, BDNF levels, and the proportion of neurons and astrocytes, see Section 2.5.1), we considered as the highest concentration 37.1 μM (equal to IC_20_), performing 1-to-1.15 serial dilutions.

### 2.3 Characterization of NSC metabolic competence by quantitative real-time PCR (qPCR) analyses

Analysis of gene expression by qPCR was performed in hiPSC-derived NSCs undergoing differentiation upon single and repeated treatment with 21 μM CPF or solvent control (0.1 % DMSO), collecting samples after 1, 3, 6 and 24 h (as shown in Fig. 2). RNA was isolated using the RNAqueous®-Micro Kit (ThermoFisher) according to manufacturer's instructions, and 500 ng of total RNA was reverse transcribed by using the High Capacity cDNA Reverse Transcription Kit (as directed, ThermoFisher).

qPCR reactions were run in duplicate using TaqMan® Gene Expression Master Mix (ThermoFisher) and the TaqMan gene expression assays shown in Table 1 (all from ThermoFisher).

**Table 1:** Genes and probes ID used for qPCR analysis.

Gene name	Assay ID
CYP1A2	Hs00167927_m1
CYP2B6	Hs04183483_g1
CYP3A4	Hs00604506_m1
CYP3A5	Hs00241417_m1
CYP3A7	Hs00426361_m1
CYP1A1	Hs01054796_g1
CYP2C19	Hs00426380_m1
CYP2C9	Hs04260376_m1
CYP2D6	Hs04931916_gH
GSTM1	Hs01683722_gH
GSTT1	Hs04399358_g1
GSTM3	Hs00356079_m1
GSTA4	Hs01119249_m1
CYP2C8	Hs00946140_g1
ACTB	Hs99999903_m1
GAPDH	Hs02758991_g1

Fluorescent emission was recorded in real-time using the ABI PRISM Sequence Detection System 7900HT (ThermoFisher). PCR amplification conditions consisted of 45 cycles with primers annealing at 60 °C. Relative RNA quantities were normalized to the reference genes GAPDH and Bactin and untreated cells were used to set calibrating conditions (ΔΔCt Method). Three biological replicates were performed.

### 2.4 Kinetics analysis

#### 2.4.1 Abiotic processes

##### 2.4.1.1 Chemical solubility and stability

The solubility and stability of CPF in water, medium and DMSO (the vehicle used to prepare stock solutions), were checked in preliminary assays, mimicking the actual experimental conditions: maximum concentration used; 37 °C; controlled pH; different incubation times. Samples in amber glass vials with screw caps were stored at −80 °C until the analysis for quantifying CPF by means of HPLC-UV was carried out.

##### 2.4.1.2 Cross-contamination among wells

In the adjacent wells to those containing CPF, at the applied concentrations, a corresponding amount of DMSO (vehicle control) was added. At the end of the experiment, the content of these control wells was stored at −80 °C until HPLC analysis. The CPF content in control wells served as an indication of possible contamination across wells, due to CPF evaporation.

##### 2.4.1.3 Adsorption to plastic

Since CPF is a lipophilic chemical, its potential adsorption to the plastic wells was evaluated during the kinetic experiments. At the end of each experiment, the content (cells and medium) of each sample well was removed. Wells were washed twice with PBS, to remove possible cellular debris and 3 mL methanol (MeOH) were added. The plate was sealed with Parafilm and kept under gentle shaking at room temperature for 2h, before transferring the complete volume into amber glass vials with screw caps. The fractions were stored at −80 °C until HPLC analysis.

##### 2.4.1.4 Non-specific binding to the matrix

Due to the presence of Matrigel (Reduced Growth Factors), pre-coated well plates were treated in parallel, with the same experimental conditions of the main experiment, but in the absence of cells. In order to determine if and how CPF was sequestered by proteins in Matrigel, the content of the pre-coated wells was transferred into amber glass vials with screw caps and stored at −80 °C until HPLC analysis. For all these analyses, 4 biological replicates were carried out.

#### 2.4.2 Analysis of the kinetic CPF behaviour after treatment of NSCs

The nominal concentration causing 5 % decrease of viability (IC_5_) was previously identified, extrapolating a dose response curves obtained with six CPF nominal concentrations (0.49, 1.95, 7.81, 31.25, 125, and 500 μM) *vs* solvent control (0.1 % DMSO) after 14-day treatment by means of resazurin test (CellTiter-Blue® Reagent) [52]. The IC_5_, equal to 21 μM, was selected not to cause a marked toxicity and therefore maintaining constant the number of cells throughout the entire duration of the experiment, as previously reported [52].

CPF stock solutions were prepared in DMSO and stored in glass at −20 °C until the beginning of each experiment. Freshly prepared treatment solutions were obtained after the dilution of stock solutions in a ratio of 1:500 in serum-free culture medium, keeping DMSO concentration at 0.1 %. Aliquots of the stock and daily treatment solutions (considered as the starting solutions) were stored at −80 °C until HPLC analysis.

Cells (about 21,000 cells/cm^2^), seeded on 6 well PDL- and reduced growth factor Matrigel coated plates, were treated during the differentiation process, starting from day 7 of differentiation up to day 21, with treatment/medium refreshed twice a week (Fig. 2). Exposure to CPF of cells or pre-coated wells (without cells) was initiated by adding 2 mL per well of the corresponding treatment solutions or the vehicle (control wells). Four biological replicates were carried out.

#### 2.4.3 Sample preparation for HPLC analysis

A first set of experiments was used to fine tune the experimental conditions, including time points and exposure conditions, taking into account possible metabolism, bioaccumulation and/or physical sequestration processes. All the fractions (i.e., medium, cells, plastic and matrix-bound fractions) were collected, at the first and last day of treatment, at different time points (i.e., 1, 3, 6, 24 h) selected on the basis of the intrinsic features of the compound and the cellular model. At each time point, the medium was transferred into amber glass vials and stored at −80 °C until HPLC analysis. Cells were washed twice with PBS (2 mL/well); to avoid dehydration of the cells, an aliquot of PBS was kept in each well. PBS was then removed, cells were scraped from the wells in 250 μL MeOH, additional 100 μL MeOH were added for washing and all the content was transferred into amber glass vials. The same procedure was applied also to the wells used for measuring matrix-bound material (without cells). Cells were then sonicated until they were fully homogenized (about 15 min). All fractions, including DMSO from control wells and MeOH used to measure possible plastic binding, were stored at −80 °C until HPLC analysis. Four biological replicates were carried out.

#### 2.4.4 Quantitative analysis by HPLC-UV

All samples from kinetic experiments were analysed using high-performance liquid chromatography (HPLC), as previously described [67], to quantify CPF and its metabolites. Prior to analysis, one volume of the collected medium samples was mixed with three volumes of MeOH. All the mixtures, including cell and matrix-bound samples, were vortexed for 30 s and then centrifuged (Eppendorf 5417R; 4 °C, 2500 rpm) for 10 min. Preliminary assays were conducted to evaluate CPF and its metabolite recovery after the solvent extraction procedure from each type of sample. Recovery was almost complete (around 100 %) in all fractions for CPF and its metabolites. Plastic-bound fraction was injected as such.

The HPLC system included a PerkinElmer Series 200 analytical pump, a Restek™ Pinnacle ODS Amine C18 column, a PerkinElmer LC 235 Diode Array Detector and the PerkinElmer Totalchrom™ 3.1.2 software for data acquisition and evaluation. For analysing CPF, CPFO and TCP, a gradient elution was applied at a flow rate of 1.0 mL/min, starting at 80:20 % methanol /buffer (Tris /HCl 50 mM EDTA 1 mM pH 7.4) and reaching after 4 min a percentage of 70:30 (v/v), respectively, maintained for 16 min. Then, the gradient returned to the initial concentration before the next sample was injected. Eluate absorption was measured continuously at 245nm for CPF and CPFO, at 310nm for TCP. Calibration curves (correlation coefficient R^2^ = 0.997) were made using commercially available reference compounds to enable quantification. The coefficient of variation was around 10 % and the limit of quantification was in the range of 0.3−0.5 μM. The retention times were 3.2; 5.6; 16.8 min for TCP; CPFO; CPF, respectively.

In order to have comparable results across the four biological replicates, the obtained raw data were normalised towards the number of cells per well, which did not vary significantly among the different wells (data not shown); hence, the compound content in different compartments was expressed as nmol per well.

### 2.5 Toxicodynamic evaluation

#### 2.5.1 Immunocytochemistry (IC) and high content imaging (HCI)

After 14 days of treatment with CPF at the nominal concentrations of 18.45, 21.21, 24.39, 28.05, 32.26, and 37.10 μM (37.10 μM equal to IC_20_) [52], obtained considering a 1-to-1.15 dilution factor, cells were fixed with 4 % formaldehyde for 10 min and washed three times in PBS 1 × . After 15 min permeabilization with PBS 1X containing 0.1 % Triton-X-100 and 3,5 % bovine serum albumin (BSA), cells were further incubated with 3,5 % BSA and 1X PBS (blocking solution) for 15 min, to prevent nonspecific binding of antibodies, and then incubated at 4 °C overnight with primary antibodies in blocking solution. For synaptogenesis analysis, cells were stained with microtubule-associated protein-2 (MAP2, chicken, 1:3000, Abcam), synaptophysin (pre-synaptic marker) (SYP, rabbit, 1:300, Abcam), and post-synaptic density protein 95 (PSD95, mouse, 1:300, Abcam) specific antibodies. Neurite outgrowth and BDNF protein levels were assessed by staining the cells with an antibody specific for β-III-tubulin (mouse, 1:500, ThermoFisher), and one for BDNF (rabbit, 1:70, ThermoFisher). Cells were also stained for glial fibrillary acidic protein (GFAP, chicken, 1:500, Abcam). The following day, cells were washed three times with PBS 1X and incubated for 1 h at room temperature in the dark with DyLight-conjugated secondary antibodies (1:500, Abcam) and nuclei counterstained with 1 μg/mL DAPI (ThermoFisher). Mean fluorescence intensity and the relative percentages of immunocytochemically-defined cell types were quantified using the ArrayScan™ XTI High Content Platform (Cellomics) (ThermoFisher) and the ArrayScan algorithm 'Neuronal Profiling V4.1', as previously described [68,69]. This algorithm applies a specific nucleus mask around the DAPI stained nucleus, distinguishing between live and pyknotic/dead cells, a cell body mask around the cell type antibody/antigen staining (i.e., MAP2, β-III-tubulin, or GFAP). Other masks were applied to identify the neurites, their branch points, and the fluorescence intensity levels of SYP and PSD95 puncta, or BDNF protein. The Cellomics platform was set up to take a minimum of 12–16 pictures/well at 10X magnification. A total of 4–8 internal replicates for each condition were performed, considering 4 biological replicates.

#### 2.5.2 Electrophysiological measurements

Cells were plated in complete ND medium onto polyethyleneimine (PEI)- and laminin-coated 24-well-MEA plates (24-Well Plate Glass (24W300/30G-288) V.232, 12 gold microelectrodes/well) (MultiChannel Systems) (5 × 10^5^ cells/well), and on day 7 of differentiation cells were treated with CPF at 21 μM or solvent control (0.1 % DMSO) for 14 days. Electrical activity was evaluated before adding CPF (0d) or solvent control, and monitored after 3, 7, 10 and 14 days of treatment (Fig. 2). Representative electrode recordings were considered for the analysis of spike rate (number of spikes/sec), burst count (considering a burst as a train of at least 5 spikes occurring within 100 millisec), and network burst count (the number of bursts that resulted synchronized among all 12 electrodes). Electrical activity was recorded using the Multi-well MEA-System [70], considering a sampling rate of 20,000 Hz, a low-pass filter cutoff frequency of 3500 Hz, and a high-pass filter cutoff frequency of 100 Hz. Data represent the average of at least three biological replicates.

### 2.6 Data analysis

Statistical significance of data (i.e., HCI, MEA and kinetic data) was assessed by one-way ANOVA with Dunnett's Multiple Comparison Test as Post Test, comparing various conditions vs solvent control (Ctr, 0.1 % DMSO) or *vs* NSCs (undifferentiated cells), or *vs* time point 0 (0d treatment) using GraphPad Prism 5 (https://www.graphpad.com/). All data represent the average of at least three biological replicates (i.e., average of minimum 3 passages) ± standard error mean (S.E.M.). Significant differences were reported in all graphs (* p < 0.05, ** p < 0.01, *** p < 0.001).

## 3 Results

### 3.1 Analysis of cytochrome P450 (CYP) and glutathione S-transferase (GST) gene expression in control and CPF treated NSCs undergoing differentiation

**Fig. 3:**
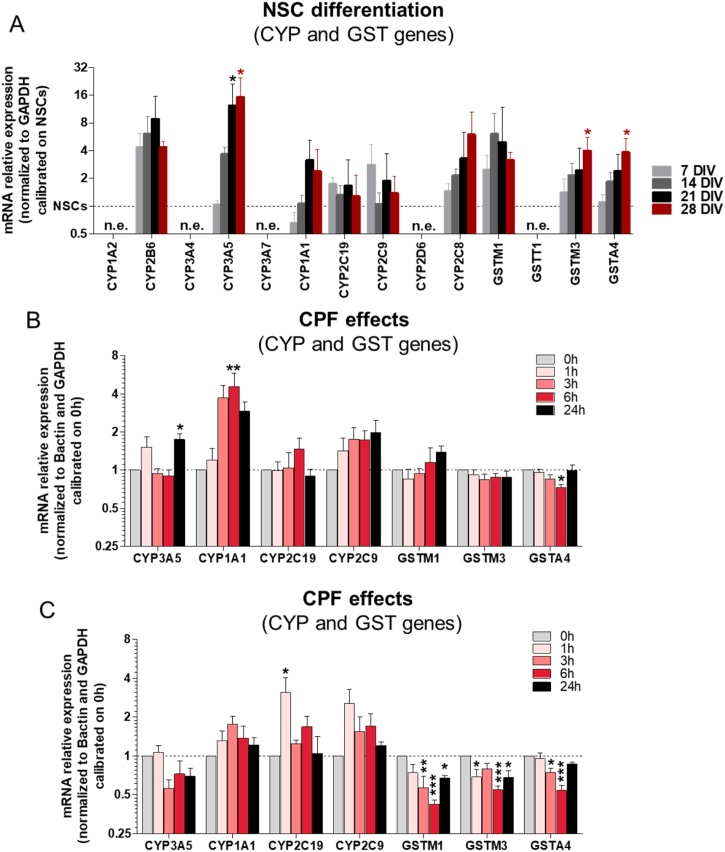
**Characterization of cytochrome P450 (CYP) and glutathione S-transferase (GST) gene expression in control culture during differentiation, and effects of CPF on CYP and GST gene expression**. (A) Bar graph shows expression of several CYP and GST genes over the course of differentiation in control culture. Analysis was carried out after 1, 7, 14, 21 and 28 days of differentiation. (B, C) Bar graphs report the expression of selected CYP and GST genes after single (B), and repeated (C) dose treatment with 21 μM CPF. Values were calibrated on NSCs (1 DIV, in A), and 0 h treatment (B and C), and normalized to GAPDH reference gene (A), and to GAPDH and Bactin genes (B, C). Data are represented as mean ± S.E.M. of 3 biological replicates. (n.e. = not expressed).

In order to evaluate the metabolic competence of hiPSC-derived NSCs undergoing differentiation towards neurons and astrocytes, we characterized the expression of several drug metabolism-related genes (Table 1). It was found that CYP-2B6, -3A5, -1A1, and -2C8 resulted upregulated upon differentiation, while CYP-2C19 and -2C9 were more modestly increased (Fig. 3A). Notably, although CYP-2B6 gene was detected via qPCR analysis, its expression level was very low and unstable across passages, with Ct values often > 40. Moreover, the expression of some glutathione S-transferase (GST) genes, able to detoxify CPFO by conjugation with reduced glutathione, was investigated and an increase over the course of differentiation in our test system was observed specifically for GST-M1, -M3, and -A4 (Fig. 3A).

Pesticides like CPF can influence both CYP and GST levels by altering the transcription of their genes [[71], [72], [73], [74]]. Therefore, we investigated the possible effects of 21 μM CPF (corresponding to IC_5_, calculated on the basis of viability (resazurin) assay, as reported in [52]) on the expression of the CYPs and GSTs involved in its metabolism and that were found more stably expressed across different passages in our cell model.

Up-regulation of CYP-1A1 gene was observed on the first day of CPF treatment, after 3 h, 6 h (p < 0.01), and 24 h (Fig. 3B). A tendency towards upregulation of CYP-2C9 was also observed on the first day of CPF treatment, while analysed GST genes did not change, apart for a modest decrease of GST-A4 observed after 6 h treatment with CPF (Fig. 3B). Upon repeated treatment, CYP genes did not significantly change over time, except for a transient upregulation of CYP-2C19 occurring after 1 h treatment (p < 0.05) (Fig. 3C). Interestingly, all analysed GST genes underwent a significant downregulation after repeated treatment with CPF (Fig. 3C).

### 3.2 Abiotic processes influencing CPF concentrations

According to the previously described step-wise procedure [21], abiotic processes (i.e., solubility and stability, cross-contamination among wells, adsorption to plastic, and non-specific physical sequestration by the matrix) possibly affecting the nominal exposure concentration were considered first (Fig. 1). The significant recovery of known amount of CPF, observed during our preliminary experiments, confirmed that under the applied experimental conditions (maximum concentration, temperature, pH and different incubation times), CPF was soluble and chemically stable in aqueous medium solutions and DMSO (Table 2).

**Table 2:** Impact of non-specific processes on the actual CPF in vitro concentrations.

Abiotic process		
Recovery from medium	108.2 ± 18.6 %	
	First day of treatment	Last day of treatment
Cross-contamination among wells	Not detectable	Not detectable
Bound-to-plastic fraction	0−8.4 %	7.5−16.5 %
Bound-to-matrigel fraction	1.7−4.2 %	5.8−6.5 %

CPF is known to extensively bind to plasma proteins and to partition into lipids [75], but the absence of serum in our culture medium justifies the complete CPF recovery from the medium (108.2 ± 18.6 %).

According to its volatility potential (vapour pressure: 2.02 × 10^−5^ mm Hg at 25 °C) [76], evaporation can occur, but preliminary experiments indicated only a negligible cross-contamination among wells.

The lipophilicity of CPF (log K_ow_, octanol/water partition coefficient = 4.96) [76], justifies the need to evaluate the plastic-binding of CPF and its metabolites over time. The obtained results evidenced adsorption to the plastic devices only by the parent compound, during the first day of treatment (range 0−8.4 %), increasing after repeated treatment (range 7.5−16.5 %) (Table 2), suggesting that this parameter should be followed during the biokinetic experiment.

Blank samples run in parallel with the main experiment, revealed that Matrigel could sequester CPF, at the same extent at the first (range 1.7−4.2 %) and the last day of exposure (5.8−6.5 %). These values (around 5 %) were considered within the experimental variability, and in view of the absence of the cells (which represents the “worst case”, since cells are thought to compete with Matrigel), this process was considered negligible.

### 3.3 CPF kinetic parameters evaluation in neuronal/glial culture derived from NSCs during 14-day treatment

The actual concentration of CPF in the medium at the beginning of the experiment (here indicated as treatment solution) was higher than expected, that is 24.5 ± 5.6 μM for a nominal concentration of 21 μM (IC_5_) with coefficients of variation (CV) of 22 %.

**Fig. 4:**
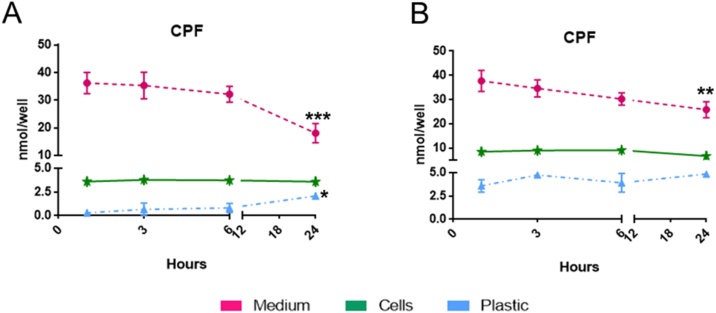
Time dependence of CPF in the medium (dashed purple line), in NSCs (solid green line) and on plastic (dotted blue line), after single (A, first day of treatment) and repeated (B, last day of treatment) exposure to 21 μM CPF. Values (nmol/well) are given as mean of four biological replicates ± S.E.M (For interpretation of the references to colour in this figure legend, the reader is referred to the web version of this article).

During the first day of treatment with IC_5_ CPF nominal concentration (corresponding to the actual one = 24.5 ± 5.6 μM), CPF was rapidly taken up: the intracellular levels of CPF measured after 1 h remained almost constant (2.5–4.7 nmol/well) until 24 h (Fig. 4A). Simultaneously, a steady state from 1 h to 6 h (36.2−32.1 nmol/well) was measured in the medium followed by a drop at 24 h (18.0 ± 6.9 nmol/well; around 41 % of the initial CPF amount) (Fig. 4A). The potential adsorption to the plastic was confirmed to be increased over time.

On the last day of treatment, the content of CPF in the medium remained stable with a drop at 24 h (37.7−25.8 nmol/well) (Fig. 4B, dashed purple curve), while in the cell compartment, the amount of CPF doubled compared to the one measured on the first day of treatment, and these differences were statistically significant (p < 0.01). Values remained almost constant over the 24 h time-period (around 8.5, 6.6 nmol/well, respectively) (Fig. 4B, green curve). Moreover, a moderate but significant change (p < 0.01), in plastic adsorption potential of CPF was noticed comparing the first and the last day of treatment (dotted blue curves, Fig. 4A and B).

**Fig. 5:**
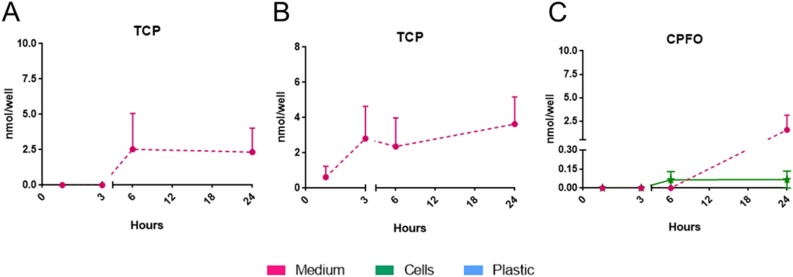
Time dependence of TCP in the medium (dashed purple line) after single (A) and repeated (B) exposure; TCP was absent in both cell and plastic fractions. Distribution of CPF-oxon (CPFO) in the medium (dashed purple line) and in NSCs (solid green line) after single exposure (C) to 21 μM CPF. Values (nmol/well) are given as mean of four biological replicates ± S.E.M (For interpretation of the references to colour in this figure legend, the reader is referred to the web version of this article).

The CPF detoxification metabolite 3,5,6-trichloro-2-pyridinol (TCP) was present only in the medium-compartment, with quite variable values on the first and the last day of treatment (about 6–18 and 7–14 %, respectively) (Fig. 5A and B). No TCP was detectable in the intracellular compartment or adsorbed to the plastic at any time point.

CPF-oxon (CPFO), the CPF toxic metabolite, was measured inside the cells during the first day of treatment after 6 h and 24 h (∼0.07 nmol/well; 0.2 %); after 24 h, a 10-time higher amount of CPFO was found in the medium (0.78 nmol/well; ∼2.9 %) (Fig. 5C). On the last day of treatment, CPFO was no longer detectable; no CPFO adsorbed to the plastic was detectable at any time point (data not shown).

**Fig. 6:**
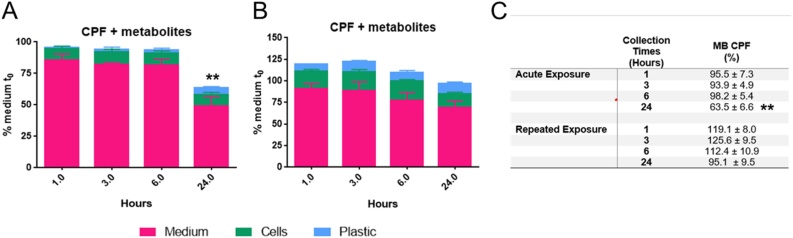
Distribution of CPF and its metabolites (CPFO and TCP) in the medium (purple bars), cell (green bars) and plastic (light blue bars) fractions at the selected collection time points after single (A) and repeated exposure (B) to 21 μM CPF, along with relative Mass Balance (C). Values (%) are given as mean of four biological replicates ± S.E.M (For interpretation of the references to colour in this figure legend, the reader is referred to the web version of this article).

For total recovery (i.e., mass balance, Fig. 6) all the different contributions (CPF, CPFO and TCP, content in all the different fractions) were considered. The mass balance was quite stable (around 95 % of the applied CPF amount) during the first three time points (1, 3 and 6 h) on the first day of treatment. A remarkable and statistically significant (p < 0.01) decrease in mass balance (around 64 %) was calculated after 24 h (Fig. 6A).

Repeated CPF exposure led to slightly increased mass balance, with values ranging from 110 to 120 % within the first 6 h, then slightly decreasing after 24 h (95.1 ± 18.9 %) (Fig. 6B).

### 3.4 CPF effects on DNT endpoints

NSCs undergoing differentiation were treated for 14 days with CPF at the nominal concentrations of 18.45, 21.21, 24.39, 28.05, 32.26, and 37.10 μM (with the highest concentration corresponding to IC_20_, as reported in [52]) in order to test its effects on the selected DNT endpoints, i.e., neurite outgrowth, number of synapses, the levels of BDNF protein, the proportion of neurons and astrocytes (assessed by means of immunocytochemistry (IC) and high content imaging (HCI)), and the generation of spontaneous electrical activity (by means of multi-well MEA).

#### 3.4.1 Neurite outgrowth, synaptogenesis and BDNF protein level

**Fig. 7:**
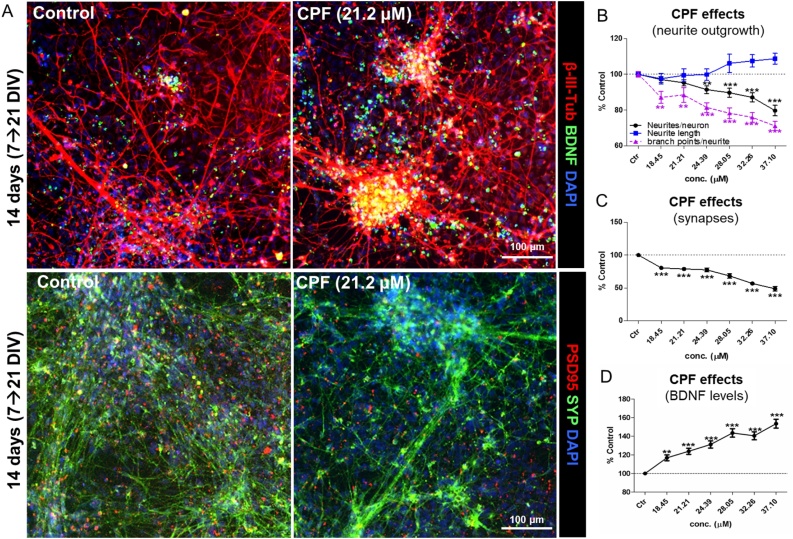
**Effects of CPF on neurite outgrowth, number of synapses and BDNF protein levels.** (A) Representative immunocytochemical images (at 10x magnification) of cells treated with solvent control (0.1 % DMSO) or CPF at 21.2 μM for 14 days, and stained for β-III-Tubulin (red) and BDNF (green) (upper panels), and PSD95 (red) and SYP (green) (lower panels). (B-D) Graphs report: neurite outgrowth-related parameters (i.e., neurite length (blue), number of neurites/neuron (black), and number of branch points/neurite (dashed purple)) (B); the number of synapses, identified by the number of overlapping SYP+/PSD95+ puncta (C); and total BDNF protein levels (D), upon treatment with low and very low toxic concentrations (≤ IC_20_) of CPF. All samples were normalised to solvent control (0.1 % DMSO, Ctr) at the respective time point. Data are represented as mean ± S.E.M. of 4 biological replicates (For interpretation of the references to colour in this figure legend, the reader is referred to the web version of this article).

Data showed that CPF at concentrations ≤ IC_20_ elicited a concentration dependent decrease in the number of neurites/neuron (Fig. 7A and B, black curve), and a decrease of neurite complexity, as shown by the lower number of branch points/neurite (Fig. 7B, dashed purple curve). However, the length of neurites was not significantly affected by CPF at these concentrations (Fig. 7B, blue curve).

Moreover, analysis of synaptophysin (SYP, pre-synaptic) and post-synaptic density protein 95 (PSD95) revealed a remarkable dose-dependent decrease of synapses (identified by the co-localization of SYP+/PSD95+ puncta) following CPF treatment (Fig. 7A, C).

Analysis of BDNF protein levels indicated a progressive dose-dependent increase upon 14 days of CPF treatment (up to ∼55 %) (Fig. 7A, D), which may be indicative of activation of a pro-survival mechanism induced by cells exposed to increasing concentrations of CPF.

#### 3.4.2 Relative proportion of neurons and astrocytes and electrical activity

**Fig. 8:**
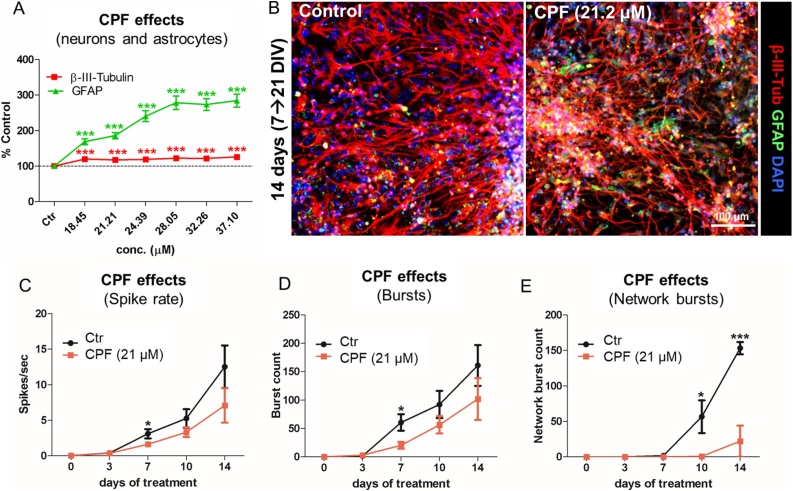
**Effects of CPF on the percentage of neurons and astrocytes and on electrical activity.** hiPSC-derived NSCs were differentiated for 7 DIV and further treated for 14 days with different concentrations of CPF. (A) Graph shows the percentage of β-III-Tubulin + neuronal cells (red), and glial fibrillary acidic protein (GFAP)+ astrocytes (green). (B) Representative immunocytochemical images (at 10x magnification) of cells treated with solvent control (0.1 % DMSO) or CPF at 21.2 μM for 14 days, and stained for β-III-Tubulin (red) and GFAP (green). (C-E) Graphs report analysis of spontaneous electrical activity in cells undergoing differentiation towards neurons and astrocytes and treated with either solvent control (0.1 % DMSO, black curve) or 21 μM CPF (orange curve). Spike rate (number of spikes/sec) (C), the total number of bursts occurring within 5 min recording (D), and the network burst count (i.e., the number of synchronized bursts occurring within 5 min recording) (E) were analyzed respectively after 0, 3, 7, 10 and 14-day treatment with CPF or 0.1 % DMSO. All samples were normalised to medium containing solvent only (0.1 % DMSO). Data are represented as mean ± S.E.M. of 4 biological replicates (For interpretation of the references to colour in this figure legend, the reader is referred to the web version of this article).

We further investigated the effects of CPF on the percentage of neuronal cells and astrocytes, assessed by means of β-III-Tubulin and glial fibrillary acidic protein (GFAP) immune-staining, respectively. CPF induced a modest but significant increase (10–15 %) of neuronal cell percentage with all tested concentrations (Fig. 8A and B, red). Interestingly, a remarkable concentration dependent increase of GFAP^+^ cell percentage was also observed (Fig. 8A and B, green) (almost 2- to 2.8-fold increase *vs* solvent control).

Analysis of spontaneous electrical activity recorded after 0, 3, 7, 10 and 14-day treatment with either solvent control (0.1 % DMSO) or CPF at 21 μM (IC_5_), showed a modest decrease of both spike rate and the number of bursts induced by CPF; in particular, this decrease was statistically significant (p < 0.05) after 7-day treatment (Fig. 8C and D). Importantly, starting from the 10th day of CPF treatment, a decrease in the number of synchronized network bursts, indicative of mature neuronal network formation, was observed (Fig. 8E).

## 4 Discussion

The assessment of chemical effects on DNT relevant endpoints anchored to common KEs described in the AOP network should encompass also biokinetic evaluation, in order to increase reliability of in vitro data and better reflect in vivo dose-response toxicity.

The relevance of DNT has been evidenced by the association between exposure to environmental pollutants and neurodevelopmental disorders in children (e.g., learning disabilities, autism spectrum disorders, attention deficit hyperactivity disorder) [[77], [78], [79]], which are becoming increasingly prevalent in recent years.

Exposure to the organophosphate insecticide CPF in infants and children has been shown by the presence of CPF in human breast milk as well in children’s urine [[80], [81], [82], [83], [84]] and considered of concern. In addition, prenatal exposure to CPF has been demonstrated by the detection of CPF in umbilical cord blood [85], and of its metabolites, such as diethylphosphate (0.80–3.20 μg/g) and diethylthiophosphate (2.0–5.6 μg/g) in human postpartum meconium, the intestinal content of the foetus [86].

Although still under debate, prenatal exposure to CPF has been associated with: (i) cerebral structural abnormalities that included thinning of the cerebral cortex, as shown by MRI analysis [31,87]; (ii) long-lasting changes in spatial learning and memory formation in juvenile and adult rats [88]; and (iii) decrease in cholinergic presynaptic markers in the hippocampus, midbrain, striatum, brainstem and cerebral cortex in juvenile and young adult rats [89]. Through non-cholinesterase mechanisms, CPF has been shown to alter synaptogenesis, neuronal network formation and BDNF signalling in adult zebrafish brain tissues [90], in differentiating PC12 cells in vitro [91], in young rats [92], and to cause inhibition of neurite outgrowth in PC12 cells [93], and in primary cultures of embryonic rat sympathetic neurons [94].

Our data show that a repeated treatment with CPF at concentrations ≤ IC_20_ decreased the number of neurites/neuron and branch points/neurite (indicative of compromised neurite outgrowth) in a concentration dependent manner, which could contribute to the observed decrease of synapse formation. These effects ultimately impacted neuronal network formation, leading to a decrease of electrical activity and neuronal network functionality, with a remarkable reduction of synchronized network bursts occurring after 10-day treatment with CPF at IC_5_, which is in line with previous studies [95,96]. Additionally, a concentration dependent increase of BDNF protein levels was observed upon treatment with CPF, confirming our previous study [52]. Along the same line, BDNF upregulation has been shown to occur also in the hippocampus and cerebral cortex of young rats exposed to CPF [92].

BDNF is known to control neuronal plasticity, long-term potentiation and depression, survival, differentiation, and final maturation [97]. Interestingly, astrocytes have been shown to express BDNF receptor (TrkB) at high levels, in particular during morphological maturation [98]. Astrocytes have also been shown to express and release BDNF, although at a lower extent than neurons [99,100], which suggests that astrocytes may contribute to regulation of BDNF availability to neurons [101]. The observed increase of astrocytes coupled with the increase of BDNF protein levels, are possibly indicative of activation of neuronal survival mechanisms prompted by astrocytes upon exposure to CPF in this mixed culture of neurons and astrocytes.

In order to adjust the nominal concentrations that triggered the above described effects, we analysed all the processes possibly affecting CPF bioavailability in our cellular model, considering adopting the necessary measures (e.g., use of glass whenever possible) to minimise any possible interference. The lipophilicity of CPF, CPFO and TCP (log K_ow_ = 4.8, 2.89 and 3.10, respectively), estimated by predictive models (EPI Suite™ [102]), are in line with the measured adsorption of CPF to plastic devices increasing over time, similarly to what obtained with more lipophilic compounds (e.g., amiodarone, log K_ow_ = 7.6 [103]), but not of its more hydrophilic metabolites. Such results confirm that K_ow_ values around 4 can provide indications about the need of measuring adsorption to the plastic, which should then be performed on a case-by-case basis. Another key factor affecting bioavailability is the protein or lipid binding of the test compound to culture medium proteins and lipids; indeed, CPF has a high affinity for human plasma proteins [104], which affect CPF free concentration and possible metabolism [105]. Since the cell model used in this study does not require the use of serum, our in vitro conditions cannot reflect the actual in vivo situation. However, considering the difference between protein binding in vivo and in vitro, the obtained in vitro results could be used for in vitro-in vivo extrapolation (QIVIVE), as it has been done with other substances [106]. In our previous study [52], we considered as in vitro nominal LOAEC 21 μM CPF (corresponding to an IC_5_) specific for the analysis of synaptogenesis upon 14-day treatment. Based on the data reported in the present study, it would be possible to refine the LOAEC, by considering the intracellular CPF concentration over the 14-day treatment and defining an in vitro point of departure (PoD). However, the design of specific PBPK modelling enabling an appropriate QIVIVE, as well as the application of a benchmark dose (BMD) model could be used to define the actual in vivo PoD, considering also the shape of the entire in vitro concentration-response curve and variability.

Some recent computer simulations have been developed to predict the in vitro distribution of various chemicals under different experimental conditions [107,108]. They can represent an approximation for predictions in non-proliferating cell systems with low metabolic capacity or stable compounds, but they are not fully suitable for metabolically competent test systems treated with chemicals undergoing biotransformation. This is even more relevant for those chemicals, such as CPF, requiring metabolic activation to elicit critical biological effects, and the assumption of low or almost absent metabolic clearance can lead to overestimation of intracellular concentrations of the parent molecule, neglecting the role of active metabolites, and therefore diverging significantly from the in vivo situation.

For these reasons, in this study, for the first time, the metabolic competence of hiPSC-derived NSCs undergoing differentiation toward a mixed population of neurons and astrocytes has been characterized, as well as the in vitro kinetics of CPF and its relevant metabolites.

For the analysis of kinetics, we selected CPF concentrations in the range of those suggested as the most appropriate to mimic in vivo conditions [109] and representative of human exposure (10−20 μM) [110], or exposure in new-borns (e.g., 23.6 μM in meconium [111], and up to 4.9 μM in blood [112,113]). These levels were estimated by applying pharmacokinetic models to biomonitoring data in adults, children, pregnant women and new-borns [75,111,112,[114], [115], [116]]. Moreover, these concentrations were found to have a low toxicity (IC_5_) throughout the repeated treatment, allowing maintaining the number of cells constant, as requested for the kinetic experiments, but sufficient to induce potential adverse effects on neurodevelopmental endpoints, as previously reported [52]. Other in vitro studies have considered CPF concentrations in the range of 10–150 μM to investigate the mechanisms underlying CPF-mediated neurotoxicity (e.g., [91,[117], [118], [119]]). Pregnancy-PBPK models would help predict the actual levels of CPF reaching the developing brain and its possible accumulation over long-term exposure.

The time-course of in vitro CPF kinetics, described here, clearly shows that CPF rapidly enters the cells, and after repeated treatment, no relevant bioaccumulation of the parent compound was evidenced, as shown by the estimation of the overall mass balance (Fig. 6C). The overall CPF kinetic profile indicated that mixed neuronal/glial cells obtained from hiPSC-derived NSCs exhibit modest metabolic competence, as confirmed by analysis of some drug metabolism-related gene expression. It is interesting to note that, on the first day, CPFO was detectable inside the cells after 6 h. CPFO at toxicologically relevant concentrations has been shown to impair neuronal proliferation, apoptosis, neuronal and glial cell differentiation [120], in line with our study. Oxon-dependent inhibition of neuronal and glial cell differentiation has been shown to be up to 1000-times greater than that caused by their parent compound [120]. The presence of CPFO at least on the first day of treatment, although at low levels, can be associated with its direct interaction with receptors and cell signalling molecules important in neurodevelopment and morphogenic activity shown by acethylcolynesterase (AChE). These effects can occur when the inhibition of AChE enzymatic activity is yet not evident [120].

Following the time course of CPFO, our results showed that its content increases in the medium after 24 h; in line with this, CPF is well-known to undergo cellular detoxification via extrusion of CPFO by membrane-bound transporters P-glycoprotein (PgP) [121]. The expression of PgP by endothelial cells and perivascular astrocytes of the BBB in human new-borns has been demonstrated [122], indicating this as a possible mechanism of extrusion of toxic compounds during the developmental stages in humans. However, the mechanism underlying the efflux is rather complex, implying active transport and passive permeability at the same time [123].

In our study, repeated treatment with CPF elicited an increase in the percentage of astrocytes (by 2–2.8 fold) in the mixed neuron-astrocyte co-culture. Recent findings have shown that the co-culture of astrocytes and neurons potentiates CPF detoxification pathway; CPF treatment was found to increase GFAP levels in primary human foetal astrocytes [124], and to increase the expression of both microglia and astrocytes in the substantia nigra of newborn rats [125]. An inhibition of neurite length, neurite number and branch points was observed in neuron-only cultures, starting at 10 μM [126], whilst in astrocyte-neuron co-cultures, astrocytes were found to protect neurons from the effects of CPF at higher concentrations, with astrocytes effectively metabolizing CPF through endogenous CYP 450 enzymes activation [126].

Some CYPs (-1A1, -2B6, -2C19, -2C8, -2C9 and -3A5) and GSTs (A4, -M1 and -M3) have been found expressed in our hiPSC-derived cell model undergoing differentiation towards neurons and astrocytes. Notably, brain CYPs are regulated through transcriptional, post-transcriptional and post-translational mechanisms [127]. The level of CYPs in the brain is approximately 0.5–2 % of that found in liver, being detectable generally with highly sensitive techniques, such as qPCR. This suggests that brain CYPs may not have significant impact on the overall pharmacokinetics of drugs and hormones in the body [128]. However, *in situ* metabolism can be relevant for those chemicals or their metabolites toxic to brain cells or for drugs acting on the CNS. At the same time, brain CYPs play also an important role in regulating the levels of endogenous GABAA receptor agonists, modulating brain cholesterol homeostasis and retinoids levels. With respect to what is reported regarding CYPs in human brain in vivo, we could not detect the expression of two major CYPs, namely i) CYP-2D6, which, on the contrary, has been detected in the brain, mainly in pyramidal neurons (in layers III–V), and in white matter [129], where it may control the biosynthesis of dopamine and 5-hydroxytryptamine [130,131]; ii) CYP-3A4, which has been found expressed in human hippocampal pyramidal neurons [132] by some anti-epileptic drugs (e.g., oxcarbazepine, carbamazepine and phenytoin). CYP-3A4 has been linked to the metabolism of testosterone and estradiol [133,134], influencing mood, behaviour, sexuality, memory and cognition [135]. The lack of CYP-1A2 is not surprising, being mainly a hepatic form.

Among the CYP isoforms detected in our test system, CYP-2B6 has been found expressed in different human brain regions [129], such as the frontal cortex. In particular, astrocytes surrounding cerebral blood vessels (in layer I) have been found to express CYP-2B6 at high levels, which suggests that this CYP isoform may influence the chemical form (parent or metabolites) entering the brain [136]. CYP-2B isoforms in the human brain modulate the sensitivity to centrally acting drugs (such as the anaesthetic propofol), affecting drug response and behaviour [127].

CYP-2C19 has been found expressed in the human brain, where it modulates the metabolism of testosterone and progesterone, controlling reward dependence, cooperativeness and other personality traits [137,138]; CYP-1A1 controls calcium signaling, vesicle release and cerebral arteries vasodilation, by mediating the metabolism of arachidonic acid in the brain [128,139].

We did not investigate the expression of other CYPs that have been detected in the human foetal brain, such as CYP-2R1 and -4X1, which have been found expressed, along with CYP-1A1, in late-term human brain tissues, but at lower levels than the corresponding adult tissues [140]. Also CYP-26A and -26B have been found highly expressed in both foetal [141] and adult human brain [142], while CYP-46 has been found expressed in the adult human brain (specifically in caudate nucleus and putamen) [143].

With regard to GST enzymes, GST alpha and pi proteins have been detected in the tela chorioidea in the telencephalon, and GST pi (but not alpha) has been found in the pia mater of the telencephalon, as shown in a human embryo at 8 week gestational age [144]. By comparing adult and human foetal brains, Carder et al. found that GST alpha was expressed only in the adult brain (mainly in choroid plexus, vascular endothelium, ventricular lining cells, pia-arachnoid and astrocytes), while GST pi was found highly expressed in the foetal brain (from as early as 12 weeks of gestation) mainly in tanycytes and in radial glia cell bodies, while neurons resulted consistently negative [145].

Based on current knowledge, at concentrations (about 10−20 μM), similar to the one used in the present study, the human CYPs mainly involved in in vitro CPF activation to CPFO in the liver are CYP-1A2 and CYP-2B6. CPF dearylation into TCP is mediated mainly by CYP-2C19, while at higher concentrations (∼100 μM, typical of accidental acute intoxication or poisoning episodes) CYP-3A4 shows a higher activity for both desulfuration and dearylation [67,109].

Since CYP-3A4 is not constitutively expressed in our in vitro model and CYP-1A2 is typically expressed in the liver, other CYP isoforms, e.g., CYP-1A1, -3A5, -2B6 found expressed in our test system, might compensate, being responsible for CPF bioactivation. On the other hand, CYP-2C19 may form TCP in hiPSC-derived NSCs undergoing differentiation towards neurons and astrocytes. In line with our results, it has been shown that CYP-1A1, which is highly expressed in human foetal tissue and and the brain, is mainly present in neurons and astrocytes, as reported in a 3D embryonic rat cellular model [140,146]. CYP-1A1 can be upregulated by the exposure to CPF in primary human hepatocytes [71], which supports our results, showing a tendency towards upregulation of CYP-1A1 gene after single treatment with CPF (Fig. 3B).

The lack of CPFO detection during the last day of treatment could be attributed to the decreased or unstable expression of involved isoforms (as in the case of CYP-2B6) over the 14 days of treatment, but can also be due to its further metabolism, by unspecific esterases or conjugation reactions, e.g., by GST, considered as additional detoxification pathways [109,147,148]. Some GST genes (-M1, -M3 and -A4) are expressed in our cell model at levels increasing with time of differentiation .

After repeated treatment with CPF, a significant decrease of GST gene expression was observed (Fig. 3C), suggesting an impairment of cellular detoxification capacity, which could contribute to the DNT effects observed after prolonged CPF treatment (decrease of synapses, neurite outgrowth and spontaneous electrical activity) (Figs. 7,8).

Exposure to CPF has been shown to increase the level of GST-A1 mRNA in HepG2 cells [73]. On the other hand, CPF exposure was also found to reduce GST activity in Zebrafish larvae [72], and to downregulate GST gene expression in the brain of Cyprinus carpio L. [74]. However, the metabolism of CPFO should be further investigated in our test system, in order to assess how the low metabolic rate paralleled with a very limited bioaccumulation observed in our experimental conditions.

The presence of TCP only in the medium is not surprising, suggesting that it gets extruded rapidly after its formation. Indeed, TCP is known as a detoxifying metabolite that is excreted in the urine (along with other metabolites), and thus used as a biomarker for organophosphorus pesticide exposure in humans [149,150]. However, a robust comparison between the levels of metabolites measured in our in vitro study and the ones observed in vivo will be feasible only by applying an appropriate extrapolation possibly achievable by using a specific PBPK model, by taking into account both liver and in situ metabolism [150,151].

TCP formation in our test system is likely linked to CYP-2C19 expression; CYP-2B6 could also play a role, at least during the first day of treatment, although its expression was unstable in our cell model. CYP-2B6 has been previously reported to be highly expressed in human astrocytes [129], and the levels of TCP have been shown to increase in astrocytes upon exposure to CPF for 2 days, both when cultured alone and in co-culture with neurons differentiated from human PSCs [126].

Although it is considered a detoxification metabolite with respect to AChE inhibition, Zurich and colleagues have reported that TCP may exert toxic effects specifically on astrocytes, compromising their neuroprotective function and exacerbating CPF neurotoxicity [152]. Overall, these studies suggest that astrocytes may have a dual role: on one hand, they elicit neuronal protective/detoxifying effects and, under specific conditions, they may also contribute to CPF (and other organophosphates) induced neurotoxicity.

Due to the age-related sensitivity to CPF and the potential DNT effects associated with CPF exposure during pregnancy/postnatal periods, the biotransformation in different age groups has been extensively investigated [153]. The overall analysis of the available data, i.e., in vivo data derived from immature and adult rats repeatedly exposed to CPF and CPFO [154], in vitro data showing different efficiency in metabolism of both CPF and CPFO in human hepatic microsomes [155], as well as specific PBPK models for CPF, accounting for human life-stage parameters (i.e., change of physiology and metabolism in relation to the age) and pregnancy-related changes [150], confirmed that age-dependent sensitivity to CPF effects can be attributed, at least in part, to toxicokinetic differences [156,157].

In conclusion, the development of a battery of in vitro DNT test methods based on the use of human cells suitable to capture perturbation of key neurodevelopmental processes and signalling pathways described as key events in the DNT AOPs, should encompass also the assessment of biokinetics/toxicokinetics of chemicals under investigation. In line with this, kinetics with *ad hoc* in silico modelling to support an appropriate reverse dosimetry [107] should be regarded as part of a tiered testing strategy for DNT evaluation, to infer a more reliable in vivo PoD.

## Authorship contributions

Participated in research design: E.D.C.; F.P.; A.B.P.; E.T.

Conducted experiments: E.D.C.; F.P.; E.M.

Performed data analysis: E.D.C.; F.P.

Wrote or contributed to the writing of the manuscript: E.D.C.; F.P.; A.B.P; E.T.

## Declaration of Competing Interest

The authors have no conflicts of interest to declare.
